# 4,4′-Oxybis{*N*-[(*E*)-quinolin-2-yl­methyl­idene]aniline}

**DOI:** 10.1107/S1600536811012955

**Published:** 2011-04-13

**Authors:** Daoud Djamel, Douadi Tahar, Haffar Djahida, Hammani Hanane, Chafaa Salah

**Affiliations:** a Laboratoire d’Électrochimie des Matériaux Moléculaires et Complexes (LEMMC), Département de Génie des Procèdes, Faculté de Technologie, Université FERHAT ABBAS – SETIF, 19000, Algeria

## Abstract

The title Schiff base compound, C_32_H_22_N_4_O, was prepared by a reaction of 4,4′-diamino­diphenyl ether and 2-quinoline­carboxaldehyde. The mol­ecule consists of two 4-{*N*-[(*E*)-quinolin-2-yl­methyl­idene]amino}­phenyl units linked by an oxygen bridge. The dihedral angles between two benzene rings and between the two quinoline ring systems are 53.81 (7) and 42.56 (4)°, respectively. Inter­molecular C—H⋯N hydrogen bonding is present in the crystal structure.

## Related literature

For the biological and pharmacological activity of quinolines and their derivatives, see: Kidwai *et al.* (2000 ▶); Souza (2005 ▶); Musiol *et al.* (2006 ▶); Gómez-Barrio *et al.* (2006 ▶); Vinsova *et al.* (2008 ▶); Jain *et al.* (2005 ▶); Chen *et al.* (2006 ▶). For applications of Schiff base compounds formed by aromatic diamine and a quinoline­aldehyde, see: Izatt *et al.* (1995 ▶); Kalcher *et al.* (1995 ▶); Gilmartin & Hart (1995 ▶); Ahamad *et al.* (2010 ▶); Negm *et al.* (2010 ▶). For related structures, see: Girija *et al.* (2004 ▶); Gowda *et al.* (2007 ▶). For the synthesis, see: Issaadi *et al.* (2005 ▶); Ghames *et al.* (2006 ▶); Kaabi *et al.* (2007 ▶).
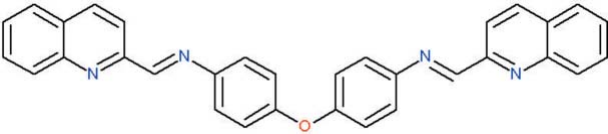


## Experimental

### 

#### Crystal data

C_32_H_22_N_4_O
*M* *_r_* = 478.54
Monoclinic, 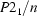

*a* = 17.4533 (7) Å
*b* = 5.0836 (2) Å
*c* = 26.817 (1) Å
β = 92.839 (1)°
*V* = 2376.43 (16) Å^3^
*Z* = 4
Mo *K*α radiation
μ = 0.08 mm^−1^
*T* = 293 K
0.25 × 0.05 × 0.05 mm

#### Data collection

Bruker APEXII diffractometer
20425 measured reflections
5473 independent reflections
4143 reflections with *I* > 2σ(*I*)
*R*_int_ = 0.035

#### Refinement

*R*[*F* ^2^ > 2σ(*F* ^2^)] = 0.042
*wR*(*F* ^2^) = 0.150
*S* = 1.1
5473 reflections
334 parameters
H-atom parameters constrained
Δρ_max_ = 0.31 e Å^−3^
Δρ_min_ = −0.30 e Å^−3^

### 

Data collection: *APEX2* (Bruker, 2002 ▶); cell refinement: *SAINT* (Bruker, 2002 ▶); data reduction: *SAINT*; program(s) used to solve structure: *SHELXS97* (Sheldrick, 2008 ▶); program(s) used to refine structure: *SHELXL97* (Sheldrick, 2008 ▶); molecular graphics: *ORTEP-3 for Windows* (Farrugia, 1997 ▶); software used to prepare material for publication: *WinGX* (Farrugia, 1999 ▶).

## Supplementary Material

Crystal structure: contains datablocks I, global. DOI: 10.1107/S1600536811012955/xu5181sup1.cif

Structure factors: contains datablocks I. DOI: 10.1107/S1600536811012955/xu5181Isup2.hkl

Additional supplementary materials:  crystallographic information; 3D view; checkCIF report

## Figures and Tables

**Table 1:** Hydrogen-bond geometry (Å, °)

*D*—H⋯*A*	*D*—H	H⋯*A*	*D*⋯*A*	*D*—H⋯*A*
C28—H28⋯N3^i^	0.93	2.57	3.434 (2)	156

Symmetry code: (i) 
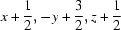
.

**Fig. 1.**
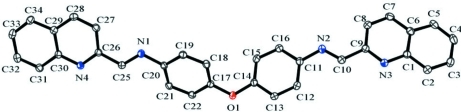
The title molecule with displacement ellipsoids for non–H atoms drawn at the 50% probability level.

**Fig. 2.**
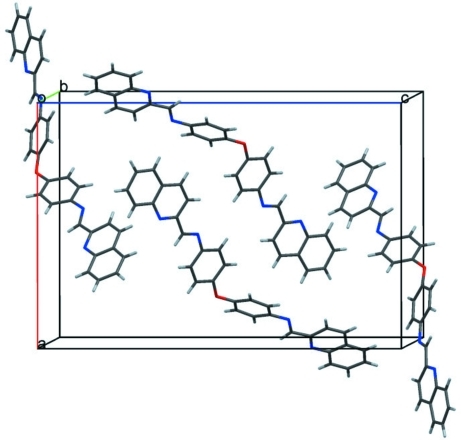
Packing of the molecules along the b–axis.

## supplementary crystallographic information

### Comment

Quinolines and their derivatives are often used for designing of many synthetic compounds with diverse pharmacological and medicinal proprieties. Literature survey reveled that substituted quinolines possess diverse chemotherapeutic activities such as antibacterial (Kidwai *et al.*, 2000), antimalarial (Souza *et al.*, 2005), antifungal (Musiol *et al.*, 2006), antiparasitical (Gómez-Barrio *et al.*, 2006), antimycobacterial (Vinsova *et al.*, 2008), antileishmanial (Jain *et al.*, 2005), and anti-inflammatory behavior (Chen *et al.*, 2006). Schiff base compounds are typically formed by condensation of an aromatic diamine and a quinolinealdehyde. These kinds of compounds have a wide variety of applications in many fields. For example in water treatment, they have a great capacity for complexation of transition metals (Izatt *et al.*, 1995; Kalcher *et al.*, 1995; Gilmartin *et al.*, 1995). They also serve as intermediates in certain enzymatic reactions and their use as corrosion inhibitors, (Ahamad *et al.*, 2010; Negm *et al.*, 2010) reveal their importance.

The compound, C_32_H_22_N_4_O prepared is a condensation product of quinolinealdehyde with bifunctional aromatic diamine as shown in Fig (1). All the molecule is found in a single asymmetric unit although, the two 4-{N-[(E) -quinolin-2-ylmethylidene] amino}phenyl moieties are related by a pseudo mirror plane. A dihedral angle of 53.15° is found between the planes defined as (O(1)—C(17)—C(18)—C(19)—C(20)—C(21)—C(22) and O(1)—C(11)—C(12)—C(13)—C(14)—C(15)—C(16). Whereas the dihedral angle between each imine phenyl plane and the attached quinolinecarboxy plane are 8.33° for C(10)—N(2)—C(11) and 17.74° for C(25)—N(1)—C(20). The bond lengths N(2)—C(10), N(2)—C(11), O(1)—C(14).. and bond angles C(10)—N(2)—C(11), C(16)—C(11)—N(2), C(9)—N(3)—C(1), N(3)—C(1)—C(2) of one 4-{N-[(E) -quinolin-2-ylmethylidene] amino}phenyl moiety are similar the corresponding ones N(1)—C(25), N(1)—C20), O(1)—C(17).. and C(25)—N(1)—C(20), C(19)—C(20)—N(1), C(26)—N(4)—C(30, N(4)—C(30)—C(31)..of the second 4-{N-[(E) -quinolin-2-ylmethylidene] amino}phenyl. The bond distances shown in table 3 indicate that the N(1)—C(25) imine (C=N) bond length of 1.268 (17) A agree with similar double bond usually observed in related compounds (Girija *et al.*, 2004) but much shorter than single C—N 1.4175 (16) A of N(1)—C(20) (Gowda *et al.*, 2007).

### Experimental

The studied Schiff base compound was synthesized in proper literature (Issaadi *et al.*, 2005; Ghames *et al.*, 2006; Kaabi *et al.*, 2007). by reacting the mixture of 4,4'-diaminodiphenyl ether (0.4 mg, 0.002 mol) and 2-quinolinecarboxaldehyde (0.64 mg, 0.004 mol) in 20 ml of boiling ethanol for 5 h, after completion of the reaction the separated solid was filtered, washed with alcohol, and finally recrystallized from ethanol and dried under vacuum. The single crystals suitable for X-ray analysis were obtained by slow evaporation from ethanol-dichloromethane (1:1).

### Refinement

H atoms were included in geometric positions C—H = 0.93 Å and refined by using a riding model [*U*_iso_ (H) = 1.2 *U*_eq_ (C)].

### Crystal data

**Table:**

C_32_H_22_N_4_O	*D*_x_ = 1.338 Mg m^−^^3^
*M**_r_* = 478.54	Melting point: 491 K
Monoclinic, *P*2_1_/*n*	Mo *K*α radiation, λ = 0.71073 Å
*a* = 17.4533 (7) Å	Cell parameters from 5947 reflections
*b* = 5.0836 (2) Å	θ = 2.3–27.4°
*c* = 26.817 (1) Å	µ = 0.08 mm^−^^1^
β = 92.839 (1)°	*T* = 293 K
*V* = 2376.43 (16) Å^3^	Needle, colourless
*Z* = 4	0.25 × 0.05 × 0.05 mm
*F*(000) = 1000	

### Data collection

**Table:**

Bruker APEXII diffractometer	4143 reflections with *I* > 2σ(*I*)
Radiation source: Enraf–Nonius FR590	*R*_int_ = 0.035
graphite	θ_max_ = 27.5°, θ_min_ = 1.4°
CCD rotation images, thick slices scans	*h* = −22→22
20425 measured reflections	*k* = −6→6
5473 independent reflections	*l* = −34→34

### Refinement

**Table:**

Refinement on *F*^2^	Primary atom site location: structure-invariant direct methods
Least-squares matrix: full	Secondary atom site location: difference Fourier map
*R*[*F*^2^ > 2σ(*F*^2^)] = 0.042	Hydrogen site location: inferred from neighbouring sites
*wR*(*F*^2^) = 0.150	H-atom parameters constrained
*S* = 1.1	*w* = 1/[σ^2^(*F*_o_^2^) + (0.0834*P*)^2^ + 0.4121*P*] where *P* = (*F*_o_^2^ + 2*F*_c_^2^)/3
5473 reflections	(Δ/σ)_max_ < 0.001
334 parameters	Δρ_max_ = 0.31 e Å^−^^3^
0 restraints	Δρ_min_ = −0.30 e Å^−^^3^

### Special details

**Table:**

Geometry. All e.s.d.'s (except the e.s.d. in the dihedral angle between two l.s. planes) are estimated using the full covariance matrix. The cell e.s.d.'s are taken into account individually in the estimation of e.s.d.'s in distances, angles and torsion angles; correlations between e.s.d.'s in cell parameters are only used when they are defined by crystal symmetry. An approximate (isotropic) treatment of cell e.s.d.'s is used for estimating e.s.d.'s involving l.s. planes.
Refinement. Refinement of *F*^2^ against ALL reflections. The weighted *R*-factor *wR* and goodness of fit *S* are based on *F*^2^, conventional *R*-factors *R* are based on *F*, with *F* set to zero for negative *F*^2^. The threshold expression of *F*^2^ > σ(*F*^2^) is used only for calculating *R*-factors(gt) *etc*. and is not relevant to the choice of reflections for refinement. *R*-factors based on *F*^2^ are statistically about twice as large as those based on *F*, and *R*- factors based on ALL data will be even larger.

### Fractional atomic coordinates and isotropic or equivalent isotropic displacement parameters (Å^2^)

**Table:**

	*x*	*y*	*z*	*U*_iso_*/*U*_eq_	
N1	0.44063 (7)	0.2230 (2)	0.11249 (4)	0.0237 (3)	
N4	0.57723 (7)	−0.2658 (2)	0.14573 (4)	0.0227 (3)	
O1	0.32016 (6)	0.7878 (2)	−0.05150 (4)	0.0302 (3)	
C25	0.49844 (8)	0.0726 (3)	0.11071 (5)	0.0239 (3)	
H25	0.5257	0.0662	0.0818	0.029*	
N3	−0.04347 (7)	1.8407 (3)	−0.14217 (5)	0.0282 (3)	
C26	0.52279 (8)	−0.0916 (3)	0.15378 (5)	0.0221 (3)	
N2	0.04205 (8)	1.3301 (3)	−0.06988 (5)	0.0294 (3)	
C30	0.60189 (8)	−0.4232 (3)	0.18480 (5)	0.0220 (3)	
C14	0.25109 (8)	0.9214 (3)	−0.05263 (5)	0.0242 (3)	
C22	0.39182 (8)	0.4332 (3)	−0.01897 (5)	0.0250 (3)	
H22	0.3994	0.382	−0.0517	0.03*	
C21	0.42483 (8)	0.2911 (3)	0.02053 (5)	0.0250 (3)	
H21	0.4545	0.144	0.0143	0.03*	
C18	0.33675 (9)	0.7328 (3)	0.03893 (6)	0.0259 (3)	
H18	0.3079	0.8823	0.045	0.031*	
C11	0.11440 (9)	1.2017 (3)	−0.06541 (5)	0.0258 (3)	
C9	−0.04232 (9)	1.6533 (3)	−0.10785 (5)	0.0263 (3)	
C13	0.24528 (9)	1.1256 (3)	−0.08705 (6)	0.0271 (3)	
H13	0.2869	1.1686	−0.1058	0.033*	
C6	−0.18146 (9)	1.8825 (3)	−0.13310 (6)	0.0269 (3)	
C12	0.17775 (9)	1.2639 (3)	−0.09323 (6)	0.0304 (4)	
H12	0.1741	1.4008	−0.1162	0.036*	
C20	0.41394 (8)	0.3668 (3)	0.06980 (5)	0.0216 (3)	
C16	0.12138 (9)	0.9950 (3)	−0.03153 (6)	0.0281 (3)	
H16	0.0798	0.9508	−0.0128	0.034*	
C17	0.34752 (8)	0.6517 (3)	−0.00961 (5)	0.0234 (3)	
C29	0.57228 (8)	−0.4008 (3)	0.23301 (5)	0.0235 (3)	
C10	0.03217 (9)	1.5231 (3)	−0.09934 (6)	0.0298 (3)	
H10	0.0738	1.5847	−0.1163	0.036*	
C31	0.65914 (9)	−0.6133 (3)	0.17642 (6)	0.0264 (3)	
H31	0.6786	−0.6303	0.1449	0.032*	
C19	0.36957 (8)	0.5879 (3)	0.07820 (5)	0.0247 (3)	
H19	0.3618	0.6396	0.1108	0.03*	
C34	0.60200 (9)	−0.5671 (3)	0.27190 (6)	0.0278 (3)	
H34	0.5836	−0.5531	0.3038	0.033*	
C28	0.51373 (9)	−0.2130 (3)	0.23956 (6)	0.0269 (3)	
H28	0.4925	−0.1939	0.2705	0.032*	
C1	−0.11244 (9)	1.9575 (3)	−0.15479 (5)	0.0260 (3)	
C33	0.65745 (9)	−0.7475 (3)	0.26262 (6)	0.0311 (4)	
H33	0.6767	−0.8554	0.2883	0.037*	
C27	0.48872 (9)	−0.0606 (3)	0.20035 (5)	0.0258 (3)	
H27	0.4499	0.062	0.204	0.031*	
C15	0.18910 (9)	0.8532 (3)	−0.02517 (6)	0.0290 (3)	
H15	0.1928	0.7139	−0.0027	0.035*	
C5	−0.25113 (9)	2.0034 (3)	−0.15027 (6)	0.0315 (4)	
H5	−0.2971	1.9519	−0.1371	0.038*	
C32	0.68574 (9)	−0.7717 (3)	0.21458 (6)	0.0300 (4)	
H32	0.723	−0.8972	0.2088	0.036*	
C7	−0.17651 (9)	1.6876 (3)	−0.09530 (6)	0.0345 (4)	
H7	−0.2201	1.6375	−0.0792	0.041*	
C8	−0.10793 (10)	1.5743 (3)	−0.08275 (6)	0.0337 (4)	
H8	−0.1041	1.4464	−0.058	0.04*	
C4	−0.25106 (10)	2.1942 (4)	−0.18585 (6)	0.0369 (4)	
H4	−0.297	2.2737	−0.1966	0.044*	
C2	−0.11438 (10)	2.1559 (3)	−0.19178 (6)	0.0364 (4)	
H2	−0.0692	2.207	−0.2061	0.044*	
C3	−0.18194 (11)	2.2725 (4)	−0.20663 (6)	0.0400 (4)	
H3	−0.1825	2.4047	−0.2306	0.048*	

### Atomic displacement parameters (Å^2^)

**Table:**

	*U*^11^	*U*^22^	*U*^33^	*U*^12^	*U*^13^	*U*^23^
N1	0.0243 (6)	0.0236 (6)	0.0232 (6)	0.0024 (5)	−0.0005 (5)	0.0004 (5)
N4	0.0221 (6)	0.0231 (6)	0.0229 (6)	0.0018 (5)	0.0005 (5)	0.0003 (5)
O1	0.0309 (6)	0.0349 (6)	0.0252 (5)	0.0122 (5)	0.0048 (4)	0.0085 (5)
C25	0.0242 (7)	0.0250 (7)	0.0226 (7)	0.0017 (6)	0.0025 (6)	0.0008 (6)
N3	0.0258 (7)	0.0310 (7)	0.0280 (7)	0.0043 (5)	0.0030 (5)	0.0047 (5)
C26	0.0203 (7)	0.0224 (7)	0.0234 (7)	−0.0010 (6)	0.0000 (5)	0.0003 (6)
N2	0.0285 (7)	0.0292 (7)	0.0304 (7)	0.0052 (6)	0.0017 (5)	0.0042 (6)
C30	0.0205 (7)	0.0206 (7)	0.0247 (7)	−0.0018 (6)	−0.0021 (5)	0.0005 (5)
C14	0.0255 (7)	0.0241 (7)	0.0228 (7)	0.0043 (6)	−0.0008 (6)	−0.0010 (6)
C22	0.0237 (7)	0.0290 (8)	0.0224 (7)	0.0025 (6)	0.0027 (6)	−0.0012 (6)
C21	0.0235 (7)	0.0236 (7)	0.0279 (7)	0.0065 (6)	0.0022 (6)	−0.0018 (6)
C18	0.0273 (8)	0.0205 (7)	0.0299 (8)	0.0053 (6)	0.0031 (6)	−0.0003 (6)
C11	0.0259 (8)	0.0257 (7)	0.0255 (7)	0.0023 (6)	−0.0012 (6)	0.0002 (6)
C9	0.0282 (8)	0.0269 (8)	0.0239 (7)	0.0035 (6)	0.0017 (6)	0.0011 (6)
C13	0.0269 (8)	0.0304 (8)	0.0243 (7)	0.0027 (6)	0.0038 (6)	0.0042 (6)
C6	0.0275 (8)	0.0260 (7)	0.0273 (7)	0.0022 (6)	0.0017 (6)	−0.0055 (6)
C12	0.0334 (9)	0.0290 (8)	0.0288 (8)	0.0052 (7)	0.0020 (6)	0.0085 (6)
C20	0.0195 (7)	0.0211 (7)	0.0242 (7)	0.0004 (6)	0.0009 (5)	0.0016 (5)
C16	0.0270 (8)	0.0285 (8)	0.0289 (8)	0.0007 (6)	0.0032 (6)	0.0040 (6)
C17	0.0216 (7)	0.0238 (7)	0.0248 (7)	0.0011 (6)	0.0007 (5)	0.0051 (6)
C29	0.0234 (7)	0.0222 (7)	0.0246 (7)	−0.0041 (6)	−0.0015 (6)	0.0002 (6)
C10	0.0263 (8)	0.0336 (8)	0.0297 (8)	0.0025 (7)	0.0027 (6)	0.0050 (7)
C31	0.0260 (7)	0.0262 (7)	0.0271 (7)	0.0012 (6)	0.0014 (6)	−0.0007 (6)
C19	0.0262 (7)	0.0245 (7)	0.0235 (7)	0.0018 (6)	0.0024 (6)	−0.0025 (6)
C34	0.0302 (8)	0.0290 (8)	0.0237 (7)	−0.0046 (6)	−0.0030 (6)	0.0028 (6)
C28	0.0291 (8)	0.0295 (8)	0.0223 (7)	0.0003 (6)	0.0043 (6)	−0.0020 (6)
C1	0.0280 (8)	0.0276 (7)	0.0226 (7)	0.0038 (6)	0.0015 (6)	−0.0011 (6)
C33	0.0318 (8)	0.0283 (8)	0.0322 (8)	−0.0019 (7)	−0.0090 (6)	0.0072 (6)
C27	0.0246 (7)	0.0260 (7)	0.0269 (7)	0.0036 (6)	0.0025 (6)	−0.0019 (6)
C15	0.0299 (8)	0.0271 (8)	0.0301 (8)	0.0029 (7)	0.0019 (6)	0.0092 (6)
C5	0.0255 (8)	0.0334 (8)	0.0359 (8)	0.0066 (7)	0.0033 (6)	−0.0085 (7)
C32	0.0261 (8)	0.0252 (8)	0.0380 (9)	0.0036 (6)	−0.0047 (7)	0.0002 (6)
C7	0.0284 (8)	0.0351 (9)	0.0410 (9)	0.0015 (7)	0.0128 (7)	0.0036 (7)
C8	0.0348 (9)	0.0325 (8)	0.0345 (8)	0.0055 (7)	0.0088 (7)	0.0111 (7)
C4	0.0349 (9)	0.0431 (10)	0.0317 (8)	0.0172 (8)	−0.0071 (7)	−0.0092 (7)
C2	0.0368 (9)	0.0402 (9)	0.0328 (8)	0.0088 (8)	0.0081 (7)	0.0092 (7)
C3	0.0486 (11)	0.0412 (10)	0.0302 (8)	0.0152 (8)	0.0012 (7)	0.0089 (7)

### Geometric parameters (Å, °)

**Table:**

N1—C25	1.2686 (18)	C6—C7	1.417 (2)
N1—C20	1.4177 (18)	C6—C5	1.419 (2)
N4—C26	1.3241 (18)	C12—H12	0.93
N4—C30	1.3706 (18)	C20—C19	1.389 (2)
O1—C14	1.3828 (18)	C16—C15	1.388 (2)
O1—C17	1.3841 (17)	C16—H16	0.93
C25—C26	1.471 (2)	C29—C28	1.416 (2)
C25—H25	0.93	C29—C34	1.421 (2)
N3—C9	1.3240 (19)	C10—H10	0.93
N3—C1	1.3697 (19)	C31—C32	1.365 (2)
C26—C27	1.418 (2)	C31—H31	0.93
N2—C10	1.266 (2)	C19—H19	0.93
N2—C11	1.4213 (19)	C34—C33	1.365 (2)
C30—C31	1.416 (2)	C34—H34	0.93
C30—C29	1.420 (2)	C28—C27	1.361 (2)
C14—C15	1.383 (2)	C28—H28	0.93
C14—C13	1.389 (2)	C1—C2	1.414 (2)
C22—C17	1.384 (2)	C33—C32	1.408 (2)
C22—C21	1.384 (2)	C33—H33	0.93
C22—H22	0.93	C27—H27	0.93
C21—C20	1.398 (2)	C15—H15	0.93
C21—H21	0.93	C5—C4	1.361 (2)
C18—C19	1.386 (2)	C5—H5	0.93
C18—C17	1.387 (2)	C32—H32	0.93
C18—H18	0.93	C7—C8	1.355 (2)
C11—C16	1.391 (2)	C7—H7	0.93
C11—C12	1.400 (2)	C8—H8	0.93
C9—C8	1.415 (2)	C4—C3	1.411 (3)
C9—C10	1.467 (2)	C4—H4	0.93
C13—C12	1.375 (2)	C2—C3	1.362 (2)
C13—H13	0.93	C2—H2	0.93
C6—C1	1.416 (2)	C3—H3	0.93
			
C25—N1—C20	120.70 (13)	C28—C29—C34	123.35 (14)
C26—N4—C30	117.80 (12)	C30—C29—C34	118.97 (14)
C14—O1—C17	121.91 (11)	N2—C10—C9	122.63 (15)
N1—C25—C26	120.80 (13)	N2—C10—H10	118.7
N1—C25—H25	119.6	C9—C10—H10	118.7
C26—C25—H25	119.6	C32—C31—C30	120.01 (14)
C9—N3—C1	117.82 (13)	C32—C31—H31	120
N4—C26—C27	123.60 (13)	C30—C31—H31	120
N4—C26—C25	115.68 (13)	C18—C19—C20	121.29 (13)
C27—C26—C25	120.72 (13)	C18—C19—H19	119.4
C10—N2—C11	120.10 (14)	C20—C19—H19	119.4
N4—C30—C31	118.33 (13)	C33—C34—C29	120.17 (14)
N4—C30—C29	122.30 (13)	C33—C34—H34	119.9
C31—C30—C29	119.37 (13)	C29—C34—H34	119.9
C15—C14—O1	124.71 (13)	C27—C28—C29	119.59 (14)
C15—C14—C13	120.53 (14)	C27—C28—H28	120.2
O1—C14—C13	114.60 (13)	C29—C28—H28	120.2
C17—C22—C21	119.69 (13)	N3—C1—C2	118.23 (14)
C17—C22—H22	120.2	N3—C1—C6	122.47 (14)
C21—C22—H22	120.2	C2—C1—C6	119.28 (14)
C22—C21—C20	120.59 (13)	C34—C33—C32	120.64 (14)
C22—C21—H21	119.7	C34—C33—H33	119.7
C20—C21—H21	119.7	C32—C33—H33	119.7
C19—C18—C17	119.01 (13)	C28—C27—C26	119.00 (14)
C19—C18—H18	120.5	C28—C27—H27	120.5
C17—C18—H18	120.5	C26—C27—H27	120.5
C16—C11—C12	118.21 (14)	C14—C15—C16	119.23 (14)
C16—C11—N2	116.80 (14)	C14—C15—H15	120.4
C12—C11—N2	124.97 (14)	C16—C15—H15	120.4
N3—C9—C8	123.37 (14)	C4—C5—C6	120.38 (16)
N3—C9—C10	114.56 (14)	C4—C5—H5	119.8
C8—C9—C10	122.00 (14)	C6—C5—H5	119.8
C12—C13—C14	119.72 (14)	C31—C32—C33	120.83 (15)
C12—C13—H13	120.1	C31—C32—H32	119.6
C14—C13—H13	120.1	C33—C32—H32	119.6
C1—C6—C7	117.34 (14)	C8—C7—C6	119.76 (15)
C1—C6—C5	118.88 (14)	C8—C7—H7	120.1
C7—C6—C5	123.78 (15)	C6—C7—H7	120.1
C13—C12—C11	121.00 (14)	C7—C8—C9	119.14 (15)
C13—C12—H12	119.5	C7—C8—H8	120.4
C11—C12—H12	119.5	C9—C8—H8	120.4
C19—C20—C21	118.61 (13)	C5—C4—C3	120.57 (15)
C19—C20—N1	116.72 (13)	C5—C4—H4	119.7
C21—C20—N1	124.52 (13)	C3—C4—H4	119.7
C15—C16—C11	121.29 (14)	C3—C2—C1	120.41 (16)
C15—C16—H16	119.4	C3—C2—H2	119.8
C11—C16—H16	119.4	C1—C2—H2	119.8
C22—C17—O1	115.31 (13)	C2—C3—C4	120.44 (16)
C22—C17—C18	120.79 (13)	C2—C3—H3	119.8
O1—C17—C18	123.77 (13)	C4—C3—H3	119.8
C28—C29—C30	117.68 (13)		

### Hydrogen-bond geometry (Å, °)

**Table:**

*D*—H···*A*	*D*—H	H···*A*	*D*···*A*	*D*—H···*A*
C28—H28···N3^i^	0.93	2.57	3.434 (2)	156

Symmetry codes: (i) *x*+1/2, −*y*+3/2, *z*+1/2.
